# Combination of tofacitinib and tacrolimus therapy in refractory interstitial lung disease associated with anti-synthetase syndrome: a case series of six patients

**DOI:** 10.3389/fimmu.2026.1847006

**Published:** 2026-09-03

**Authors:** Qiuyu Fan, Wei Huang, Shaohui Song, Huiqin Yang, Ya Liu, Chao Jia, Yu Xiao, Liang Zou

**Affiliations:** 1 Department of Immunology, Wuhan No. 1 Hospital, Wuhan, China; 2 Department of Laboratory Medicine, Wuhan No. 1 Hospital, Wuhan, China; 3 Department of Medicine Imaging, Wuhan No. 1 Hospital, Wuhan, China

**Keywords:** anti-synthetase syndrome, interstitial lung disease, refractory, tacrolimus, tofacitinib

## Abstract

**Objective:**

This study aimed to assess the efficacy and safety of tofacitinib and tacrolimus (TAT) combination therapy for treating refractory interstitial lung disease (ILD) associated with anti-synthetase syndrome (ASyS-ILD).

**Method:**

Patients with refractory ASyS-ILD who were treated with TAT at Wuhan No. 1 Hospital were included in the study from January 2020 to May 2024. A retrospective analysis of the clinical data from these patients is presented.

**Result:**

A total of six patients, one male patient and five female patients, primarily presented with refractory ASyS-ILD. All patients experienced symptomatic relief following TAT therapy, which facilitated the successful tapering of glucocorticoids (GCs, 24.67 ± 17.42 *vs.* 8.34 ± 1.89, *p* = 0.004). After treatment, PGA, PGA-VAS, PhGA-VAS, and MMT-8 scores significantly improved, and diffusing capacity of carbon monoxide (DLCO) increased in all 6 patients. Moreover, the serum levels of creatine kinase (CK) and lactate dehydrogenase (LDH) were notably reduced, and inflammatory indices, including C-reactive protein (CRP), ferritin, and erythrocyte sedimentation rate (ESR), declined significantly in all patients after administration of TAT. Additionally, peripheral white blood cell and neutrophil counts were significantly reduced, while lymphocyte counts increased, with a markedly decreased neutrophil/lymphocyte ratio in patients with ASyS-ILD receiving treatment. However, during treatment, two patients developed herpes zoster, which resolved with antiviral treatment. Unfortunately, two fatalities occurred, one due to disease progression from severe *Aspergillus fumigatus* pneumonia and the other due to pancreatic cancer.

**Conclusion:**

In this series of patients with refractory ASyS-ILD, TAT therapy showed some clinical effectiveness over the short term, with an observed steroid-sparing outcome, suggesting a possible benefit. Nevertheless, larger prospective controlled studies are necessary to further assess its efficacy and safety.

## 1 Introduction

Idiopathic inflammatory myopathies (IIMs) represent a diverse group of diseases impacting the skin, skeletal muscles, lungs, and other organs. IIMs include clinical subtypes such as dermatomyositis (DM), immune-mediated necrotizing myopathy (IMNM), and anti-synthetase syndrome (ASyS). Additionally, various myositis-specific antibodies (MSA) are associated with relatively consistent clinical features.

ASyS is characterized by the presence of inflammatory myopathy, interstitial lung disease (ILD), arthritis, Raynaud’s phenomenon (RP), or mechanic’s hands (MH), along with serum anti-aminoacyl-tRNA-synthetase antibodies (anti-ARS) (1–3). Aminoacyl-tRNA synthetase serves as both the key laboratory marker and the disease trigger. At the European League Against Rheumatism and American College of Rheumatology Annual Meeting (2017), ASyS was recognized as a distinct disease, separate from other myopathies. The lung is the most commonly affected organ, typically presenting as ILD (4). In ASyS patients, ILD is often predominant at onset, with prevalence rates ranging from 63% to 100%, making it a primary determinant of prognosis (5, 6). Previous studies indicate that patients with ASyS-ILD respond better to immunomodulation than other patients with IIM-associated ILD (IIM-ILD). However, disease recurrence frequently occurs during medication tapering or discontinuation, potentially increasing the risk of pneumonia and diminishing quality of life (7–10). Thus, treatment options for ASyS-ILD remain undefined because evidence-based algorithms are lacking. Various immunosuppressants and their combinations have been explored in case reports or series, yet recurrence rates in ASyS-ILD patients range from 30% to 50% (11–13). Some reports suggest that cyclosporine or tacrolimus (TAC) may be effective for refractory ILD in ASyS patients (14), although only a limited number of patients have been studied. 

Numerous studies have highlighted the benefits of tofacitinib (TOFA) in managing patients with interstitial lung disease (ILD) related to positive anti-melanoma differentiation-associated gene 5 (MDA5) antibodies and amyopathic dermatomyositis (ADM). In an open-label trial, 18 patients with early-stage ILD who tested positive for the anti-MDA5 antibody were treated with a combination of steroids and tofacitinib. This treatment led to a higher 6-month survival rate compared with historical controls receiving conventional treatment (15, 16). Additionally, a prospective cohort pilot study demonstrated that TOFA significantly improved both immunologic and clinical outcomes in patients with dermatomyositis (DM) and ASyS (17). 

The clinical efficacy of tofacitinib and tacrolimus (TAT) in treating patients with ASyS-ILD has not been sufficiently investigated. This retrospective study presents a case series of six patients with refractory ASyS-ILD who received TAT treatment.

## 2 Methods

### 2.1 Patients

A review was conducted of six patients diagnosed with refractory ASyS-ILD at Wuhan No. 1 Hospital from January 2020 to May 2024. Informed consent was waived due to the retrospective nature of the study.

All patients were diagnosed with ASyS based on the criteria developed by Solomon et al. in 2011 (18). ILD was diagnosed based on clinical manifestations, physical examination, and high-resolution computed tomography (HRCT) abnormalities, following established guidelines (19). Refractory ASyS was characterized by either intolerance or an inadequate response to glucocorticoids (GCs) and at least one other immunosuppressive or immunomodulatory agent for more than 6 months before the study’s commencement (20).

Patients were excluded if they had myositis associated with pregnancy, concurrent malignant tumors, severe respiratory dysfunction (with forced vital capacity (FVC) ≤30% of the predicted value), or severe cardiac dysfunction (with ejection fraction ≤30% of the predicted value).

### 2.2 Evaluation of disease severity

We reviewed the clinical and demographic characteristics of the patients, focusing on the clinical manifestations of ASyS, the clinical triad (myositis, arthritis, and ILD), complications, and serologic findings. Specialized rheumatologists evaluated the patients’ muscle strength and overall disease profile. Subsequently, manual muscle testing 8 (MMT-8), physician global activity assessment (PGA), patient global activity assessment-visual analogue scale (PGA-VAS), and physician global activity assessment-visual analogue scale (PhGA-VAS) were recorded. Patients were considered to have a flare when treatment was intensified for exacerbation of myositis or ILD with an increased dose of GCs and/or the addition of a new immunosuppressant (21).

### 2.3 Assessment of ILD

Dynamic chest HRCT scans were conducted for all patients, with each image independently evaluated by an experienced radiologist and a pulmonary specialist. According to the proposed guidelines (19), HRCT findings were primarily categorized into four patterns: usual interstitial pneumonia (UIP), nonspecific interstitial pneumonia (NSIP), organizing pneumonia (OP), and diffuse alveolar damage (DAD). 

The assessment of ILD predominantly used the semi-quantitative visual scoring system introduced by Camiciottoli et al. (22). This visual evaluation comprises two scores: severity and extent. The severity score was evaluated based on five parenchymal abnormalities, reflecting the increasing severity of lung involvement, and ranged from 0 (no abnormality) to 15 (all abnormalities present). The extent score was determined by counting the number of abnormal bronchopulmonary segments, which ranged from 0 (no abnormality in any segment) to 15 (all five abnormalities in more than nine segments). The total score, ranging from 0 to 30, is the sum of the severity and extent scores. Both evaluators conducting the assessment were blinded to the clinical data and outcomes of all patients. Additionally, the assessment focused on forced vital capacity (FVC) and the diffusing capacity of carbon monoxide (DLCO).

### 2.4 Laboratory indices

The laboratory data were extracted from electronic medical records. All patients were tested for myositis-associated antibodies, including anti-MDA5, Mi, TIF1γ, NXP2, SAE1, SAE2, SSA/Ro52, Scl-70, PM-Scl100, PM-Scl75, Ku, SRP, RNA-PIII, Fibrillarin, SPR, Th/To, NOR-90, and anti-synthetase antibodies (anti-Jo-1, PL-7, PL-12, EJ, KS, HA, Zo, and OJ antibodies). They tested positive for anti-ARS antibodies using the immunoblotting assay. Clinical laboratory tests, including routine blood tests, biochemical and infectious biomarkers, were performed for each patient during diagnosis and treatment. All data were checked by two physicians.

### 2.5 Treatment

**Figure 1:**
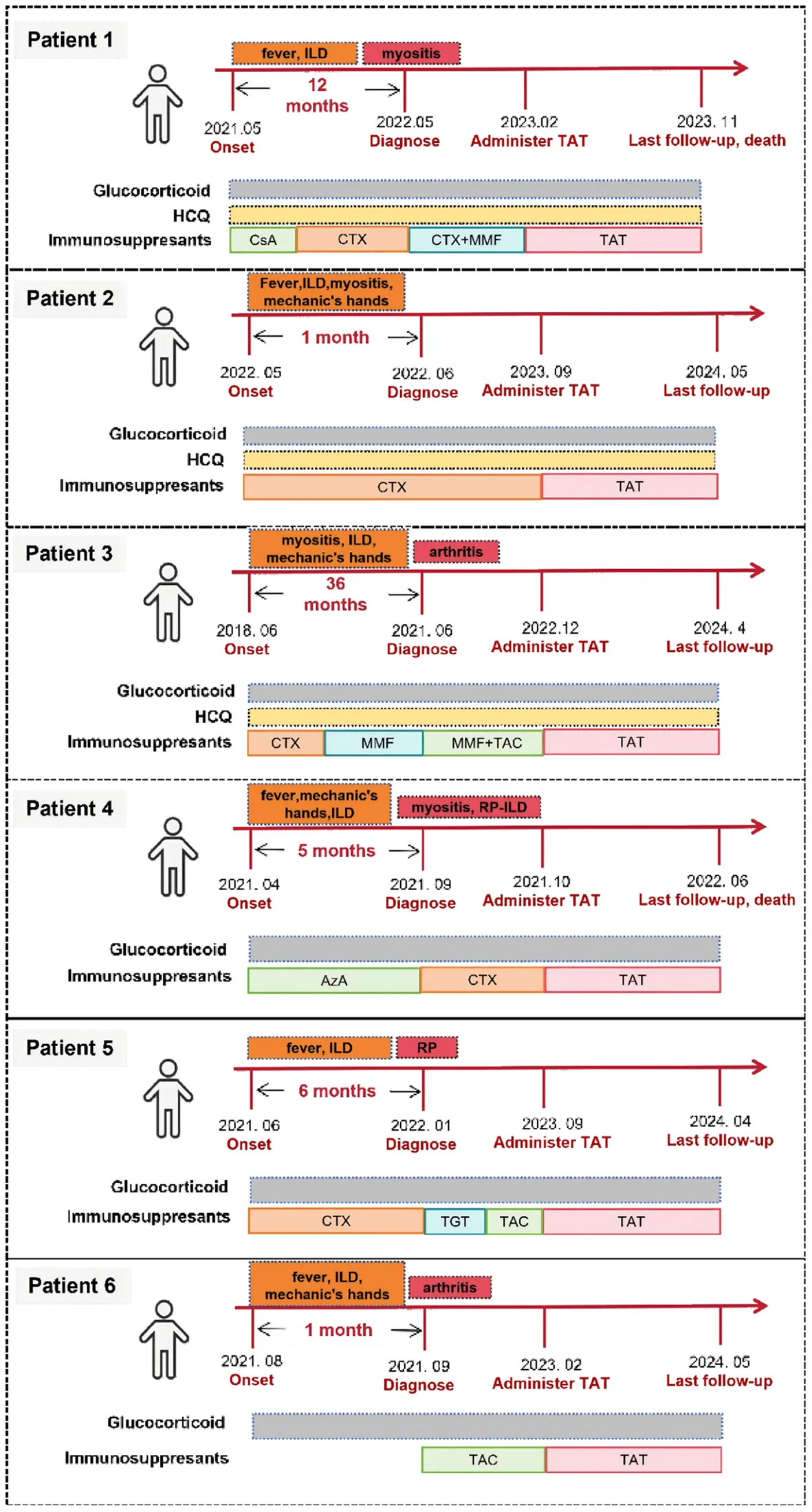
A timeline illustrating the clinical course and therapeutic process of 6 refractory ASyS-ILD patients. The timelines encompass clinical characteristics at onset, the diagnostic and therapeutic time course, treatment, and outcomes of these patients with ASyS-ILD. Orange rectangles represent the clinical manifestations at disease onset; magenta rectangles represent the clinical manifestations that appear during the disease course. CsA, cyclosporin A; CTX, cyclophosphamide; MMF, mycophenolate mofetil; TAC, tacrolimus; AZA, azathioprine; TGT, tripterygium glycosides tablet; HCQ, hydroxychloroquine.

Medications documented included systemic corticosteroids and immunosuppressants, such as cyclophosphamide (CTX), hydroxychloroquine (HCQ), TOFA, TAC, mycophenolate mofetil (MMF), and tripterygium glycosides (TGT; **Figure 1**).

Although a range of alternative immunosuppressants had been administered, all patients experienced disease recurrence during tapering of GCs (methylprednisolone-equivalent dose ≤16 mg/day), including recurrent fever or elevated CK levels due to the disease and ILD progression, ultimately necessitating the initiation of TAT therapy. TAT therapy, comprising 1 mg TAC every 12 hours and 5 mg TOFA once or twice daily, was used for each patient for at least 3 months, followed by a follow-up period of more than 6 months. During treatment, two patients developed herpes zoster. At this point, the TOFA dosage was adjusted to 5 mg/day in both patients, while TAC treatment remained unchanged. After TAT treatment, all patients underwent gradual tapering of glucocorticoids.

### 2.6 Statistical analysis

We conducted the statistical analysis using SPSS software version 22.0 (IBM Corp., Armonk, NY, USA) and GraphPad Prism 8.0 (GraphPad Software, Boston, MA, USA). Qualitative data are presented as numbers and percentages, while quantitative data are shown as the mean with standard deviation (SD) or the median with interquartile range (IQR). Safety was evaluated by reporting all adverse events.

## 3 Results

### 3.1 A timeline illustrating the clinical course and therapeutic process of patients with ASyS-ILD.

Six patients with refractory ASyS-ILD treated with TAT were identified. Among the six patients, five were female patients and one was a male patient, with a mean age at onset of 50.5 ± 13.08 years. All patients tested positive for anti-Ro52 antibodies (100%); three patients (patients 1, 3, and 4; 50%) tested positive for anti-PL-7 antibodies, two patients (patients 2 and 6; 33.3%) tested positive for anti-Jo-1 antibodies, and one patient (patient 5; 16.8%) tested positive for anti-EJ antibodies (**Table 1**). All patients experienced delays in diagnosis, with a mean time from onset to diagnosis of 10.17 ± 13.29 months, and the longest delay was 36 months (**Figure 1**).

**Table 1:** The demographic, clinical features of 6 refractory ASyS-ILD patients were presented.

Case	Age(years)/sex	First consulting department	Onset-to-TAT interval (months)	Post-TAT follow-up time (months)	MAA/MSA	Types of ILD	Previous treatment	GCs and TAT Initial dosage	GCs and TAT maintenance dosage	Before TAT	After TAT	Infection	Prognosis
1	42/female	Dermatology	21	10	anti-Ro52/ anti- PL-7 antibodies	NSIP	CTX\CsA\MMF\HCQ	GCs 20mg qd TAC 1mg q12h TOFA 5mg bid	GCs 8mg qd TAC 1mg q12h TOFA 5mg bid	HRCT-score 16 FVC(%) 70 DLCO(%) 49	HRCT-score 16 FVC(%) 68 DLCO(%) 58	NO	Death /pancreatic cancer
2	41/female	Neurology	15	9	anti-Ro52/ anti-Jo- 1 antibodies	NSIP	CTX\HCQ\TAC	GCs 16mg qd TAC 1mg q12h TOFA 5mg bid	GCs 7.5mg qd TAC 1mg q12h TOFA 5mg qd	HRCT-score 15 FVC(%) 64.2 DLCO(%) 39	HRCT-score 14 FVC(%) 65.8 DLCO(%) 51	Herpes Zoster	survival
3	46/female	Respiratory	53	17	anti-Ro52/ anti- PL-7 antibodies	NSIP/UIP	CTX\MMF\TAC	GCs 20mg qd TAC 1mg q12h TOFA 5mg bid	GCs 8mg qd TAC 1mg q12h TOFA 5mg bid	HRCT-score 26 FVC(%) 41.5 DLCO(%) 37	HRCT-score 24 FVC(%) 45 DLCO(%) 45	NO	survival
4	52/female	Respiratory	6	8	anti-Ro52/ anti- PL-7 antibodies	RP-ILD/OP	AZA\CTX\TAC	GCs 60mg qd TAC 1mg q12h TOFA 5mg bid	GCs 12mg qd TAC 1mg q12h TOFA 5mg bid	HRCT-score 28 FVC(%) 92 DLCO(%) 25	HRCT-score 24 FVC(%) 89 DLCO(%) 55	*Aspergillus fumigatus* pneumonia	death
5	46/female	Respiratory	26	8	anti-Ro52/ anti-Ej antibodies	NSIP/UIP	CTX\TGT\TAC	GCs 16mg qd TAC 1mg q12h TOFA 5mg bid	GCs 8mg qd TAC 1mg q12h TOFA 5mg qd	HRCT-score 25 FVC(%) 64.5 DLCO(%) 41	HRCT-score 22 FVC(%) 66 DLCO(%) 43	Herpes Zoster	survival
6	76/male	Respiratory	18	15	anti-Ro52/ anti-Jo- 1 antibodies	NSIP/UIP	TAC	GCs 16mg qd TAC 1mg q12h TOFA 5mg bid	GCs 6.5mg qd TAC 1mg q12h TOFA 5mg bid	HRCT-score 27 FVC(%) 71 DLCO(%) 46	HRCT-score 24 FVC(%) 112 DLCO(%) 91	NO	survival

NSIP, nonspecific interstitial pneumonia; UIP, usual interstitial pneumonia; OP, organizing pneumonia; RP-ILD, rapidly progressive interstitial pneumonia; MAA, myositis-associated antibodies; MSA, myositis-specific antibodies; MMF, mycophenolatemofetil; TAC, tacrolimus; CTX, cyclophosphamide; HCQ, hydroxychloroquine; TGT, tripterygi-um glycosides tablets; TOFA, tofacitinib; GCs, glucocorticoids (All glucocorticoid doses are presented as methylprednisolone-equivalent doses); HRCT-score, high-resolution computed tomography score; FVC, forced vital capacity; DLCO, diffusing capacity of carbon monoxide.

The clinical characteristics and treatment course are summarized below (**Figure 1**; **Table 1**):

1. Patient 1 initially presented with fever and NSIP, followed by elevated CK. Previous treatments included GCs combined with HCQ, CsA, CTX, and MMF. However, each attempt to taper GCs resulted in fever recurrence and ILD progression. After 21 months, TAT therapy (TAC 1 mg q12h, TOFA 5 mg bid) was initiated, GCs (methylprednisolone-equivalent) were gradually tapered from 20 mg/day to 8 mg/day. At the 10-month follow-up, the patient’s imaging and pulmonary function outcomes were as follows: HRCT score (16 vs. 16) and FVC (70% vs. 68%) remained stable; DLCO improved from 49% to 58%. Unfortunately, the patient died of subsequently diagnosed pancreatic cancer.
2. Patient 2 presented with fever, myositis, NSIP, and mechanic’s hands. After treatment with GCs combined with HCQ and CTX, each attempt to taper GCs also led to elevated CK and ILD progression. TAT therapy was initiated after 15 months. Soon afterward, GCs were tapered from 16 mg/day to 7.5 mg/day, while TAC was maintained at the original dose and TOFA was continued at 5 mg/day (reduced temporarily due to herpes zoster infection). At the 9-month follow-up, the patient had no fever recurrence. HRCT score decreased from 15 to 14, FVC improved slightly (64.2% vs. 65.8%), and DLCO improved from 49% to 58%.
3. Patient 3 initially presented with myositis, NSIP with UIP, and mechanic’s hands, and later developed arthritis. Previous treatments included GCs combined with CTX, MMF, and TAC. Each reduction in GCs resulted in ILD progression. After 53 months, TAT therapy was initiated, and GCs were tapered from 20 mg/day to 8 mg/day. During the 17-month follow-up period, the patient’s condition remained stable. HRCT score decreased from 26 to 24, and both FVC (41.5% *vs.* 45%) and DLCO (37 vs. 45) improved.
4. Patient 4 initially presented with fever, RP-ILD with OP, and mechanic’s hands, followed by elevated CK. After treatment with GCs combined with AZA and CTX, the patient’s rapidly progressive ILD worsened. TAT therapy was initiated at 6 months. GCs were tapered from 60 mg/day to 12 mg/day. At the 8-month follow-up, pulmonary inflammation had markedly resolved. HRCT score decreased from 28 to 24, and DLCO improved significantly from 25% to 55%. Unfortunately, the patient died of pulmonary aspergillosis.
5. Patient 5 was initially diagnosed with fever and NSIP with UIP. Previous treatments included GCs combined with CTX, TGT, and TAC. Each attempt to taper GCs resulted in fever recurrence and ILD progression. TAT therapy was initiated after 26 months. GCs were tapered from 16 mg/day to 8 mg/day, while TAC was maintained at the original dose and TOFA was continued at 5 mg/day (reduced temporarily due to herpes zoster infection). At the 8-month follow-up, the patient had no fever recurrence or ILD progression. HRCT score decreased from 25 to 22, FVC (64.5% vs. 64%) and DLCO (41% vs. 43%) improved.
6. Patient 6 initially presented with fever, NSIP with UIP, mechanic’s hands, and arthritis. Despite treatment with GCs combined with TAC, the patient experienced recurrent fever and ILD progression.

TAT therapy was subsequently initiated. GCs were tapered from 16 mg/day to 6.5 mg/day. During the 15-month follow-up period, the patient’s condition remained stable. HRCT score decreased from 27 to 24, FVC (71% vs. 112%) and DLCO (46% vs. 91%) improved significantly.

At disease onset, all patients (100%) exhibited ILD, four (66.67%) had mechanic’s hands, and five (83.33%) presented with fever (**Figure 1**). As the disease advanced, two patients (33.33%) developed arthritis, two (33.33%) experienced myalgia and muscle weakness, one (16.67%) showed Raynaud’s phenomenon, and one (16.67%) had rapidly progressive interstitial lung disease (RP-ILD) (**Figure 1**). Before TAT therapy, patients 1 and 2 were diagnosed with nonspecific interstitial pneumonia (NSIP). Patients 3, 5, and 6 progressed to NSIP along with usual interstitial pneumonia (UIP), while patient 4 deteriorated into rapidly progressive interstitial lung disease (RP-ILD) with organizing pneumonia (OP) (**Table 1**). These patients with ILD predominantly exhibited inflammatory exudation and still require active treatment to prevent irreversible pulmonary interstitial fibrosis.

### 3.2 Improvement in ASyS severity and reduction in GC dosage after TAT treatment

**Figure 2:**
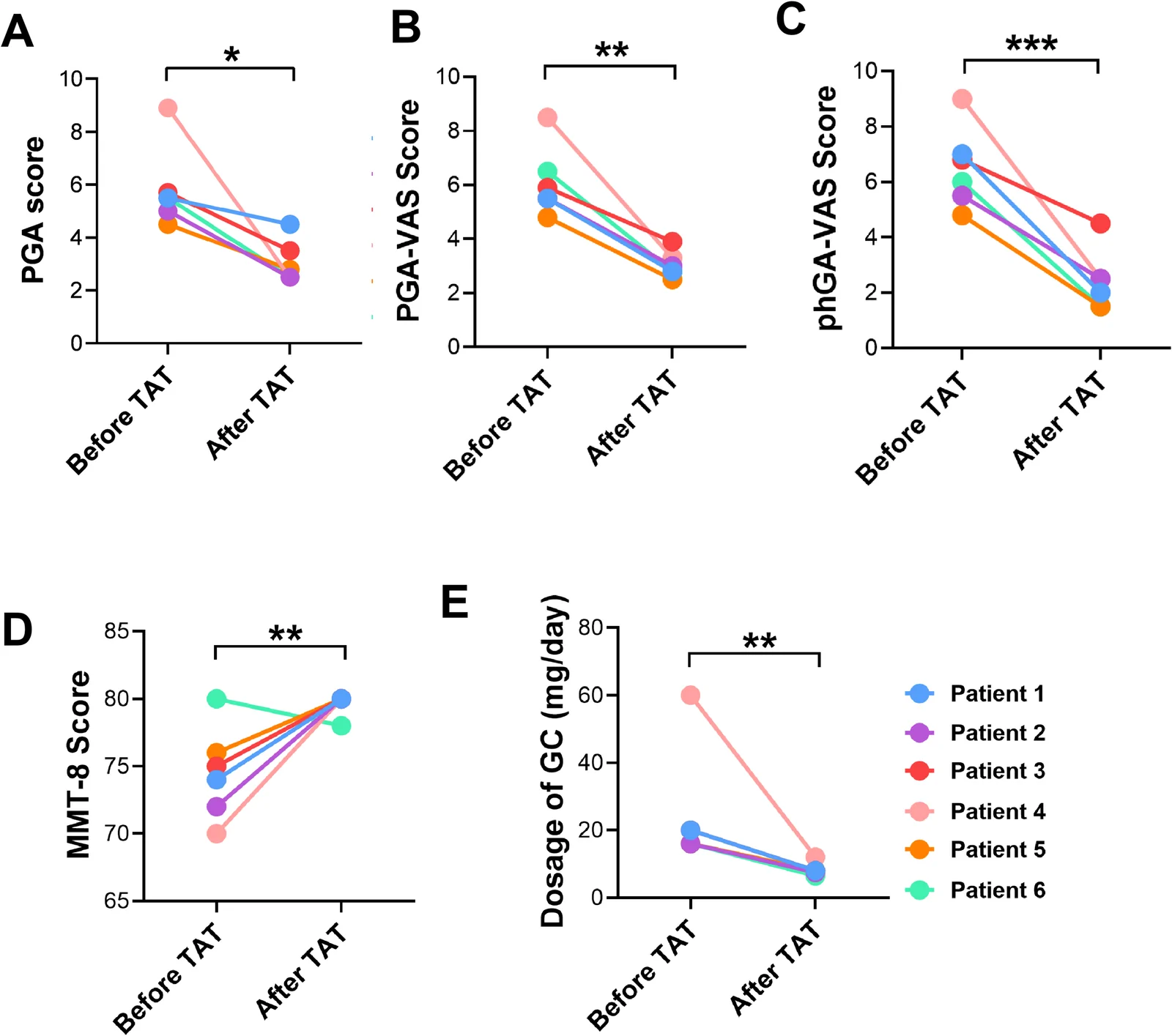
Comparison of clinical parameters of 6 ASyS-ILD patients after TAT treatment. **(A–E)** Plots show the changes in PGA, PGA-VAS, PhGA-VAS, MMT-8 score, and GC dosage of patients with ASyS-ILD before and after TAT treatment. Values are displayed as mean ± SD. *p < 0.05, **p < 0.01, ***p < 0.001.

Following TAT therapy, there was a significant improvement in MMT-8 scores (76.17 ± 4.49 *vs.* 80.00 ± 0.00, *p* = 0.005), PGA score (5.85 ± 1.56 *vs.* 3.05 ± 0.81, *p* = 0.015), PGA-VAS scores (6.12 ± 1.29 *vs.* 3.05 ± 0.49, *p* = 0.003), and PhGA-VAS scores (6.58 ± 1.73 *vs.* 3.15 ± 0.65, *p* = 0.0003), indicating a marked enhancement in patients’ quality of life (**Supplementary Table 1**; **Figure 2**). All patients showed improvement in clinical manifestations such as mechanic’s hands, myalgia, muscle weakness, and fever. All patients experienced disease recurrence during tapering of GCs (methylprednisolone-equivalent dose < 16 mg/day), ultimately requiring the initiation of TAT therapy. The initial and maintenance dosages of GCs and TAT therapy are listed in **Table 1**. After TAT treatment, the GCs dosage for all six patients was significantly reduced (24.67 ± 17.42 mg/d *vs.* 8.33 ± 1.89 mg/d, *p* = 0.004; **Supplementary Table 1**, **Figure 2**). More importantly, no disease flares were observed in any patient during TAT treatment.

### 3.3 Elevated pulmonary DLCO in patients with ASyS-ILD after TAT administration

**Figure 3:**
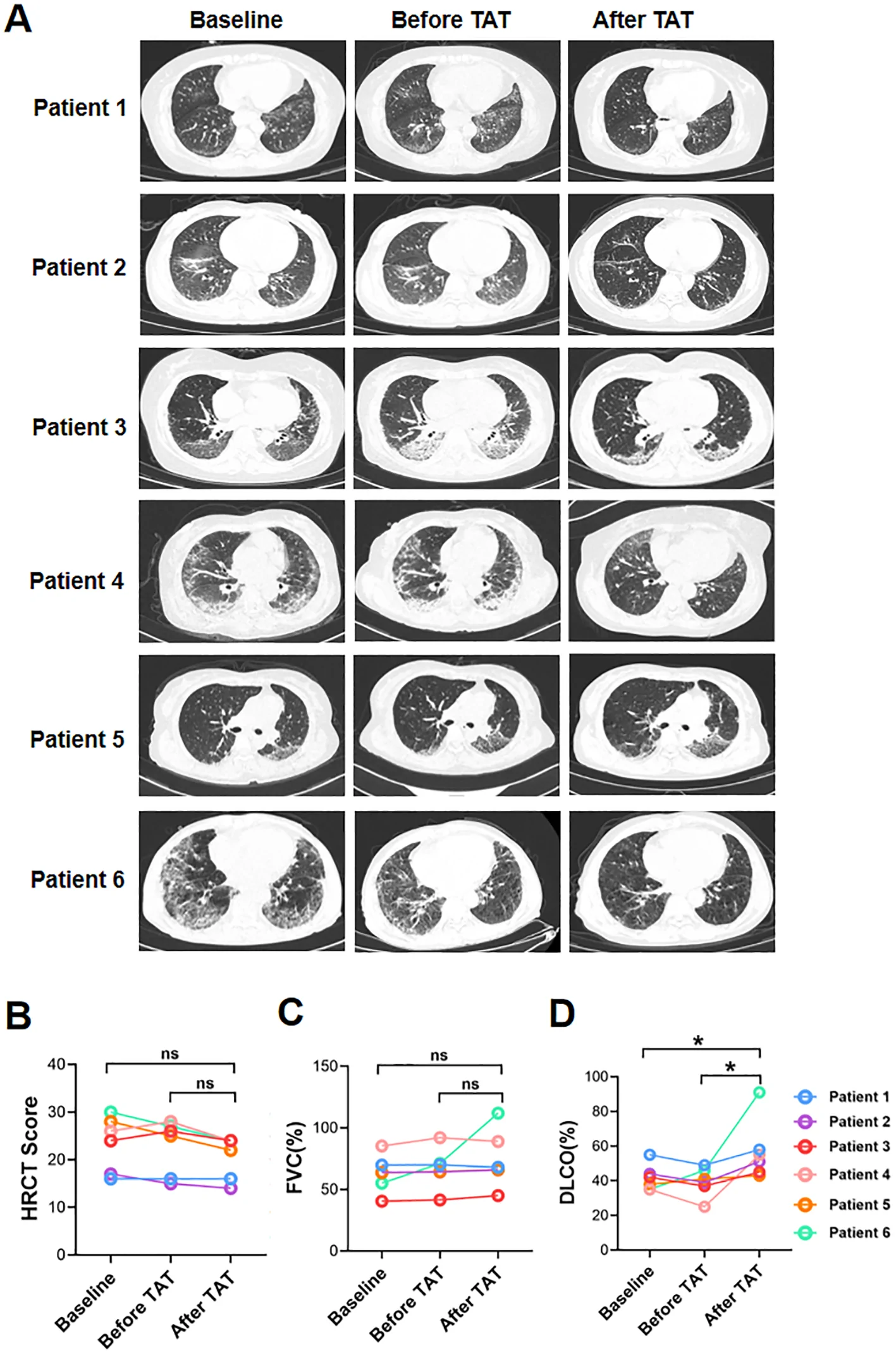
Improvement of pulmonary function in ASyS-ILD patients treated with TAT. **(A)** Imaging pictures show HRCT scans of six patients with ASyS-ILD at onset and before and after TAT treatment. **(B–D)** Plots display the changes in HRCT score, FVC (%), and DLCO (%) before and after TAT treatment. Values are displayed as mean ± SD; *p < 0.05; ns, not significant.

According to HRCT, lung imaging improved in five patients and remained unchanged in one patient (**Figure 3A**), suggesting that patients displayed no evidence of further radiographic progression of lung lesions following TAT therapy. Three patients (patients 1, 5, and 6; 50%) had moderate pulmonary diffusion impairment (DLCO), and the other three (patients 2, 3, and 4; 50%) experienced severe impairment before TAT treatment, indicating severe ILD. After TAT treatment, all patients demonstrated a significant increase in DLCO (39.5 ± 8.38% *vs.* 57.17 ± 17.53%, *p* = 0.026; **Supplementary Table 1**; **Figure 3D**). However, improvements in the HRCT score (22.83 ± 5.78 *vs.* 20.67 ± 4.50, p = 0.353) and FVC (67.2 ± 16.21% *vs.* 74.3 ± 23.14%, *p* = 0.346) were not statistically significant, although all patients showed a downward trend (**Figures 3B, C**).

### 3.4 The efficacy of TAT administration in patients with ASyS-ILD

**Figure 4:**
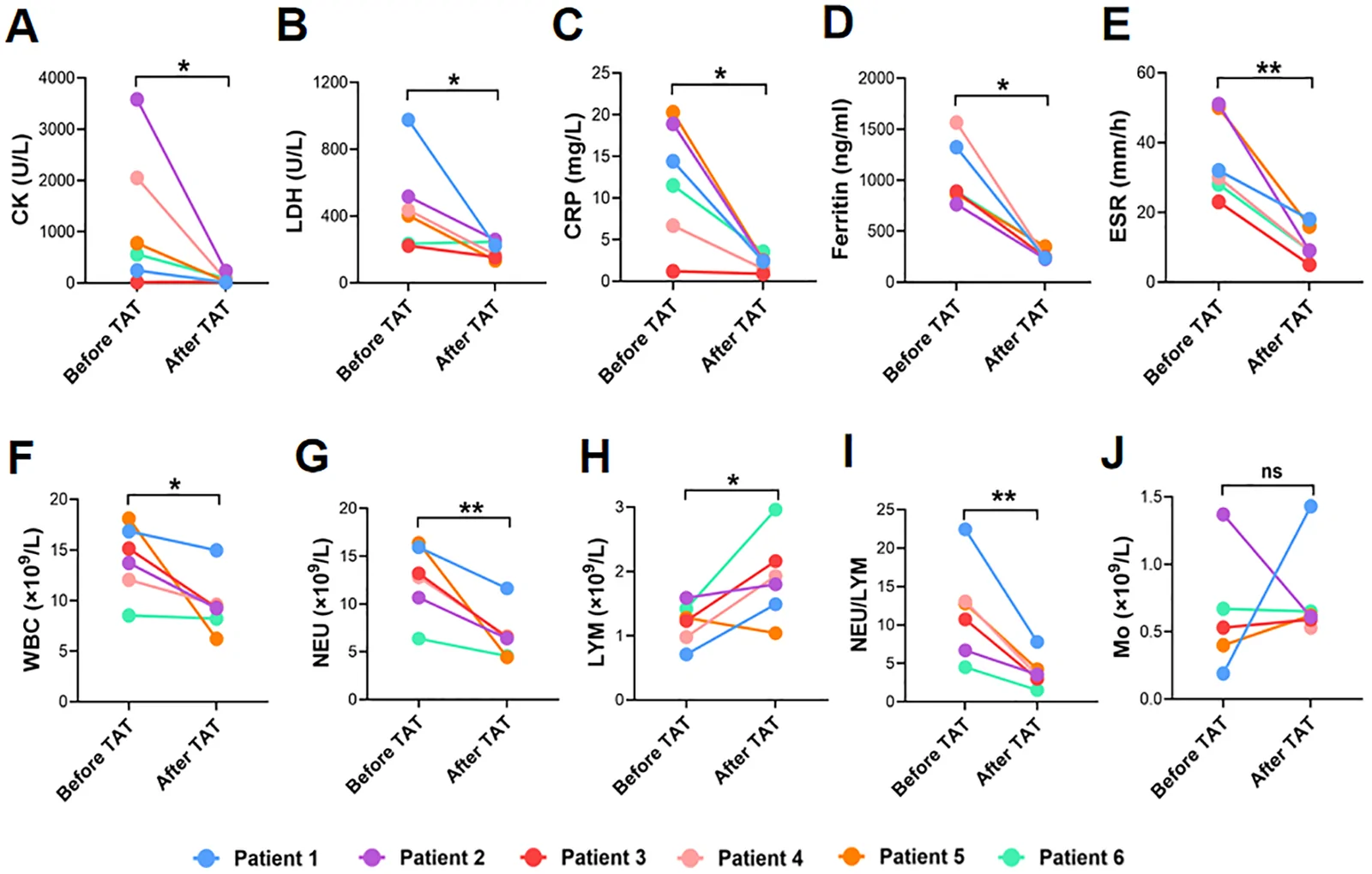
Comparison of the laboratory indicators in ASyS-ILD patients before and after TAT therapy. **(A–E)** Changes in CK, LDH, CRP, ferritin, and ESR levels in patients with ASyS-ILD after TAT treatment are shown; **(F–J)** WBC, NEU, LYM, and Mo counts and the NEU/LYM ratio in patients with ASyS-ILD are shown before and after TAT treatment. WBC, white blood cell count; NEU, neutrophil count; LYM, lymphocyte count; Mo, monocyte count; CK, creatine kinase; LDH, lactate dehydrogenase; ESR, erythrocyte sedimentation rate; CRP, C-reactive protein. Values are displayed as mean ± SD; *p < 0.05; **p < 0.01; ns, not significant.

The median follow-up duration of all patients after TAT treatment was 9.5 months (range: 8–17 months). Fortunately, following TAT treatment, the serum levels of lactate dehydrogenase (LDH; 464.50 ± 276.00 *vs.* 196.83 ± 51.86 IU/L, *p* = 0.0417) and creatine kinase (CK; 1206.00 ± 1363.30 *vs.* 74.33 ± 83.67 IU/L, *p* = 0.005) decreased in all ASyS-ILD patients (**Figures 4A, B**). Patients treated with TAT showed significantly lower peripheral white blood cell (WBC) counts (14.09 ± 3.46 × 10^9^/L *vs.* 9.59 ± 2.91 × 10^9^/L, *p* = 0.036) and neutrophil (NEU) counts (12.56 ± 3.7 × 10^9^/L *vs.* 6.68 ± 2.62 × 10^9^/L, *p* = 0.01), while lymphocyte (LYM) counts (1.20 ± 0.32 × 10^9^/L *vs.* 1.90 ± 0.65 × 10^9^/L, *p* = 0.040) increased in all patients when compared with those before treatment. Additionally, the neutrophil/lymphocyte ratio (NEU/LYM) decreased in all patients (11.71 ± 6.26 vs. 3.93 ± 2.10, *p* = 0.016) compared with before treatment. Peripheral C-reactive protein (CRP) levels (12.16 ± 7.31 mg/L *vs.* 2.22 ± 0.92 mg/L, *p* = 0.020), erythrocyte sedimentation rate (ESR; 35.67 ± 11.88 mm/h *vs.* 11 ± 4.94 mm/h, *p* = 0.003), and ferritin levels (1049.7 ± 318.94 ng/mL *vs.* 275 ± 56.09 ng/mL, *p* = 0.031) were also significantly reduced after TAT treatment compared with before treatment (**Supplementary Table 2**; **Figures 4C–J**). Furthermore, it was observed that serum alanine transaminase (ALT) and aspartate aminotransferase (AST) levels showed a downward trend in treated patients (**Supplementary Figure 1**). These results demonstrated that TAT therapy showed some clinical effectiveness in normalizing hematological CK, LDH, and inflammatory indicators in patients with refractory ASyS-ILD over a short period ranging from 8 to 17 months.

### 3.5 Clinical outcome

During TAT treatment, no prophylactic anti-infective therapy was administered, and patients 2 and 5 both developed herpes zoster, diagnosed based on clinical symptoms of unilateral segmental clustered vesicles accompanied by neuralgia. In these two patients, TOFA was temporarily discontinued for 2 weeks during the course of viral infection, while other treatments remained unchanged. Fortunately, following aggressive antiviral therapy, the symptoms resolved immediately. 2-week later, TOFA was tapered to a maintenance dose of 5 mg daily, and no further recurrence of herpes zoster was noted.

Sadly, two patients died during the follow-up period. Patient 1 died of pancreatic cancer; Patient 4 died of *Aspergillus* *fumigatus* pneumonia, confirmed by culture of alveolar lavage fluid, due to the lack of aggressive anti-infective treatment for various reasons. Patient 4 died of pancreatic cancer. Given the small sample size, no direct causal relationship between infection or mortality and TAT therapy can be established based on current evidence.

## 4 Discussion

In our retrospective study, six patients with refractory ASyS-ILD showed alleviated pulmonary inflammation and improved or stabilized ILD following TAT therapy. ILD is the most common extramuscular manifestation of ASyS, with an estimated prevalence of 67%–100%, making it a critical cause of mortality (23). Literature suggests that patients with anti-PL-7 and anti-EJ antibodies in ASyS often experience more severe and refractory ILD (24). In our study, four of six patients (66.7%) tested positive for these antibodies, aligning with the high prevalence rates documented in the literature and underscoring the clinical challenges faced by antibody-positive patients. Previous studies have shown that 78%–100% of patients with ASyS-ILD assessed by HRCT exhibit NSIP, OP, or a mixed NSIP/OP pattern (25). In our cohort, all six patients initially presented with NSIP-type ILD (100%), and one patient with anti-PL-7 antibodies progressed to RP-ILD, consistent with findings that NSIP is the most common radiological pattern in ASyS-ILD. The high proportion of NSIP in our study supports the idea that ASyS-ILD has a specific radiological presentation, aiding in early disease identification and diagnosis.

ASyS is a highly heterogeneous disease with significant variability in its early clinical manifestations. Currently, no standard treatment exists for ASyS (26). Recently, a multicenter, randomized, prospective, open-label study compared the efficacy of initial GC treatment combined with either CsA or TAC in patients with PM/DM-ILD, and the results demonstrated that, after 52 weeks, both the CsA and TAC groups displayed improved lung function compared with the GC-only group. However, no statistically significant difference was observed in progression-free survival between the two groups (27). Recently, a retrospective cohort study of 12 patients with refractory ASyS indicated that Janus kinase inhibitor (JAKi) treatment was effective for most patients and relieved patients’ symptoms, including skin rash, myositis, and ILD (20). JAKi is currently approved for the treatment of rheumatoid arthritis (RA) and psoriasis, demonstrating excellent efficacy and improving clinical remission rates (28), partly because IL-6 and IFN contribute to the pathogenesis of IIMs through the JAK/STAT pathway (29). TOFA, a pan-JAK inhibitor (JAK1/JAK2/JAK3), suppresses multiple cytokine and growth factor signaling pathways and affects downstream mediators of interferon activity. Recently, a large multicenter cohort study showed that TOFA was associated with significantly greater benefits for 1-year lung transplantation-free survival than calcineurin inhibitors in anti-MDA5-positive patients with DM-ILD (30).

Currently, no clinical studies have explored the use of TAT for treating patients with ASyS, and studies in patients with ASyS-ILD are even rarer. Our study revealed that TAT therapy significantly lowered PGA-VAS and PhGA-VAS scores in patients with refractory or recurrent ASyS-ILD who did not respond to conventional glucocorticoid and immunosuppressant therapy. Additionally, it alleviated clinical manifestations such as mechanic’s hands, myalgia, myasthenia, and fever. This suggests that TAT treatment might be associated with relief of overall disease activity and improvement in multiple ASyS-related symptoms, leading to a better quality of life. Importantly, all patients experienced a notable reduction in GC dosage, and no disease flare occurred following TAT treatment, which are crucial advantages for patients with ASyS-ILD. This not only lessens the burden of adverse effects of prolonged corticosteroid use, including osteoporosis, diabetes, and infection, but also potentially prevents disease relapse.

DLCO is the most important parameter for pulmonary function assessment and is frequently used to evaluate IIM-related ILD. In our cohort, TAT therapy improved DLCO in six patients with ASyS-ILD, while FVC did not, similar to findings reported previously (31). The treatment had a preferential effect on gas diffusion across the alveolar-capillary interface, probably through attenuation of local inflammation. This study suggests that DLCO may be a useful and sensitive indicator for assessing pulmonary inflammation improvement with TAT therapy in patients with ASyS-ILD. Additionally, HRCT scores exhibited a downward trend. Pulmonary imaging improved in five patients and remained stable in one patient, suggesting that following TAT therapy, patients displayed no evidence of further radiographic progression of lung lesions. Due to the small sample size and the severe baseline pulmonary dysfunction in patients, further research with larger cohorts is necessary to confirm the efficacy of TAT in enhancing pulmonary function parameters.

From an inflammatory marker perspective, previous studies have shown that the majority of patients with refractory ASyS experience a hyperinflammatory state, including recurrent fever and elevated markers such as ESR, CRP, and ferritin (32). Consistently, the 6 ASyS-ILD patients in our study exhibited significantly increased inflammatory markers, including ferritin, CRP, ESR, absolute white blood cell count, neutrophil count, and neutrophil/lymphocyte ratio, which were suppressed by TAT treatment. However, lymphocyte counts were elevated in patients after TAT treatment in this study, indicating enhanced anti-infective capacity. A recent report suggested that TOFA treatment inhibits T follicular helper (Tfh), Th17, and Treg cells in peripheral blood mononuclear cells (PBMCs) from patients with IIMs, improving disease activity (17). These findings align with the immunomodulatory mechanism of TOFA, further supporting its potential in regulating the dysregulated immune response in patients with IIMs.

Moreover, TAT therapy significantly lowered the serum levels of LDH and CK in patients, with especially pronounced reductions in CK levels in six patients with ASyS-ILD. This indicates that TAT might have a reparative effect on muscle damage or may help prevent further tissue injury by reducing inflammation. However, the serum ALT and AST levels showed a decreasing trend following treatment, which might be due to the limited sample size and requires further investigation.

Our study noted two cases of herpes virus infection following TAT therapy, consistent with reports documenting that JAKi treatment may increase the risk of herpes zoster, latent *Mycobacterium tuberculosis* reactivation, and other infections (33). This emphasizes the importance of closely monitoring viral reactivation risks and implementing prophylactic antiviral measures.

Additionally, two patients died during follow-up from aspergillosis pneumonia and pancreatic cancer, respectively. Notably, patients with refractory ASyS-ILD are inherently at high risk of infection due to the prolonged disease course and long-term use of moderate-to-high-dose GCs and multiple immunosuppressants. Given the small sample size and complex treatment histories, no direct relationship between infection or mortality and TAT therapy can be established based on current evidence. However, these cases underscored the need for early identification of high-risk patients, prompt aggressive treatment, and prophylactic anti-infective therapy.

To our knowledge, this is the first case series to document successful outcomes of TAT therapy in refractory ASyS-ILD. However, the small sample size and uncontrolled design of our study limit the statistical analyses and generalizability of the findings. Since all patients received moderate-to-high-dose GCs and immunosuppressants before TAT therapy, and some patients had a follow-up duration of less than 12 months, the possibility that these treatments had delayed effects on disease control cannot be excluded. Additionally, the optimal timing for initiating TAT therapy, its potential use in combination with other immunosuppressants, and the retreatment schedule require further clarification.

## 5 Conclusion

In summary, our results suggest that TAT therapy may be a viable option for treating patients with refractory ASyS-ILD. Nevertheless, the associated risk of infection must be highlighted. Larger prospective controlled studies are necessary to validate our findings.

## Data Availability

The original contributions presented in the study are included in the article/**Supplementary Material**. Further inquiries can be directed to the corresponding author.
