# Supplementary material for: Combination of tofacitinib and tacrolimus therapy in refractory interstitial lung disease associated with anti-synthetase syndrome: a case series of six patients
Source: Front Immunol. 2026 Sep 3;17:1847006. doi: 10.3389/fimmu.2026.1847006 (PMC13582451; doi:10.3389/fimmu.2026.1847006)
Supplement: Supplementary file 1 [file SupplementaryFile1.docx]

**Supplemental Table 1 Clinical characteristics of patients before and after TAT therapy**

| Characteristics | Before TAT | After TAT | *p* value |
| --- | --- | --- | --- |
| PGA Score | 5.85±1.5566 | 3.05±0.80932 | 0.015* |
| PGA-VAS Score | 6.1167±1.2937 | 3.05±0.49295 | 0.0029** |
| PhPGA-VAS Score | 6.5833±1.7256 | 3.15±0.65345 | 0.0003*** |
| MMT8 Score | 76.167±4.4907 | 80.00 ± 0.00 | 0.0051** |
| Dosage of GC (mg/day) | 24.667±17.42 | 8.3333±1.8886 | 0.0044** |
| HRCT Score | 22.833±5.7764 | 20.667±4.5019 | 0.3536 |
| FVC (%) | 67.2±16.212 | 74.3±23.137 | 0.346 |
| DLCO (%) | 39.5±8.3845 | 57.167±17.532 | 0.026* |

PGA, patient global activity assessment; PhGA, physician global activity assessment; VAS, visual analogue scale; MMT-8, manual muscle testing; GC, glucocorticoid; HRCT, high-resolution computed tomography; FVC, orced vital capacity; DLCO, diffusing capacity of carbon monoxide. Values displayed as n (%), and mean±SD. **p*<0.05, ***p*<0.01, ****p*<0.001.

**Supplemental Table 2 Clinical laboratory indexes of patients before and after TAT therapy**

| Characteristics | Before TAT | After TAT | *p* value |
| --- | --- | --- | --- |
| WBC, mean±SD | 14.058±3.4648 | 9.59±2.9087 | 0.0361* |
| NEU, mean±SD | 12.56±3.6958 | 6.6833±2.6157 | 0.0098** |
| LYM，mean±SD | 1.202±0.33065 | 1.897±0.72602 | 0.0401* |
| NEU/LYM, mean±SD | 11.705±6.2639 | 3.9297±2.1003 | 0.0163* |
| Mo, mean±SD | 0.632±0.4488 | 0.78±0.36401 | 0.631 |
| CK, mean±SD | 1206±1363.3 | 74.333±83.672 | 0.0049** |
| LDH, mean±SD | 464.5±276.04 | 196.83±51.859 | 0.0417* |
| Ferritin, ean±SD | 1049.7±318.94 | 275±56.093 | 0.0313* |
| ESR, mean±SD | 35.667±11.877 | 11±4.9396 | 0.0028** |
| CRP, mean±SD | 12.162±7.313 | 2.2167±0.91579 | 0.0204* |
| ALT, mean±SD | 105.8±110.77 | 33±30.183 | 0.103 |
| AST, mean±SD | 149.8±152.63 | 27.6±7.9246 | 0.185 |

WBC, white blood cell; NEU, neutrophil; LYM, lymphocyte; Mo, monocyte; CK, creatine kinase; LDH, lactate dehydrogenase; ESR, erythrocyte sedimentation rate; CRP, C-reactive protein; ALT, alanine transaminase; AST, aspertate aminotransferase. Values displayed as mean±SD. **p*<0.05, ***p*<0.01.

**Supplemental Figure 1**

**Supplemental Figure 1** Comparison of safty indicators in ASyS-ILD patients before and after TAT treatment. **(A-B)** Plot showing changes of alanine transaminase (ALT) and aspertate aminotransferase (AST) levels in patients with ASyS-ILD after TAT treatment. Values displayed as mean±SD.
